# Biopolymer Drug Delivery Systems for Oromucosal Application: Recent Trends in Pharmaceutical R&D

**DOI:** 10.3390/ijms25105359

**Published:** 2024-05-14

**Authors:** Natallia V. Dubashynskaya, Valentina A. Petrova, Yury A. Skorik

**Affiliations:** Institute of Macromolecular Compounds of the Russian Academy of Sciences, Bolshoi VO 31, 199004 St. Petersburg, Russia

**Keywords:** oromucosal drug delivery, buccal drug delivery, mucoadhesion, drug permeability, biopolymers

## Abstract

Oromucosal drug delivery, both local and transmucosal (buccal), is an effective alternative to traditional oral and parenteral dosage forms because it increases drug bioavailability and reduces systemic drug toxicity. The oral mucosa has a good blood supply, which ensures that drug molecules enter the systemic circulation directly, avoiding drug metabolism during the first passage through the liver. At the same time, the mucosa has a number of barriers, including mucus, epithelium, enzymes, and immunocompetent cells, that are designed to prevent the entry of foreign substances into the body, which also complicates the absorption of drugs. The development of oromucosal drug delivery systems based on mucoadhesive biopolymers and their derivatives (especially thiolated and catecholated derivatives) is a promising strategy for the pharmaceutical development of safe and effective dosage forms. Solid, semi-solid and liquid pharmaceutical formulations based on biopolymers have several advantageous properties, such as prolonged residence time on the mucosa due to high mucoadhesion, unidirectional and modified drug release capabilities, and enhanced drug permeability. Biopolymers are non-toxic, biocompatible, biodegradable and may possess intrinsic bioactivity. A rational approach to the design of oromucosal delivery systems requires an understanding of both the anatomy/physiology of the oral mucosa and the physicochemical and biopharmaceutical properties of the drug molecule/biopolymer, as presented in this review. This review summarizes the advances in the pharmaceutical development of mucoadhesive oromucosal dosage forms (e.g., patches, buccal tablets, and hydrogel systems), including nanotechnology-based biopolymer nanoparticle delivery systems (e.g., solid lipid particles, liposomes, biopolymer polyelectrolyte particles, hybrid nanoparticles, etc.).

## 1. Introduction

Improving the biopharmaceutical properties of known drugs is an attractive strategy for pharmaceutical research and development (R&D) because of its high efficacy, wide availability and low cost compared to synthesizing and commercializing completely new drugs [1]. Nanotechnology-based approach to modify active pharmaceutical ingredients is widely used to increase their solubility and bioavailability, provide targeted drug delivery directly to the diseased site, and reduce drug dosage and incidence of side effects [2,3].

One of the biopharmaceutical factors that can be used to increase the bioavailability of drugs and reduce their toxicity profile is the route of drug administration and, therefore, the type of dosage form [4,5]. For example, polymeric oral drug delivery systems (e.g., buccal tablets, patches, etc.) provide a high rate of drug absorption into the systemic circulation, reduce first-pass metabolism compared to oral dosage forms, and protect drugs from entering the aggressive environment of the gastrointestinal tract [6,7,8]. Topical oral drug delivery systems (primarily oromucosal patches) can also provide prolonged drug residence time at the desired site as well as unidirectional drug release [9,10,11]. For this purpose, polymeric drug delivery systems must have high mucoadhesion to maintain prolonged contact with the mucosa under wet oral cavity conditions, as well as provide programmed and controlled drug release and high drug absorption levels [12].

Mucoadhesive biopolymers and their derivatives (i.e., chitosan, carrageenan, hyaluronic acid, alginate, etc.) are attractive carriers for the design of drug delivery systems for oral applications with desired properties, including high efficacy and improved safety profile. These polymers are characterized by good bioadhesion to the mucosa, they enhance the paracellular transport of drug molecules, and they can be easily chemically modified. Biopolymers are non-toxic and safe, biocompatible and capable of regulated biodegradation in the body [13,14,15].

This review describes a biopharmaceutical approach to develop biopolymeric drug delivery systems for oromucosal application with high mucoadhesion and enhanced cell permeability that can improve both oral and topical drug bioavailability and reduce systemic drug toxicity. By summarizing the current status of oromucosal biopolymer drug delivery systems, the review provides a basis for researchers to identify gaps and potential areas for further research. This may have implications for the development of innovative drug-delivery technologies and novel therapeutic approaches.

## 2. Biological Barriers in the Oral Cavity

**Figure 1:**
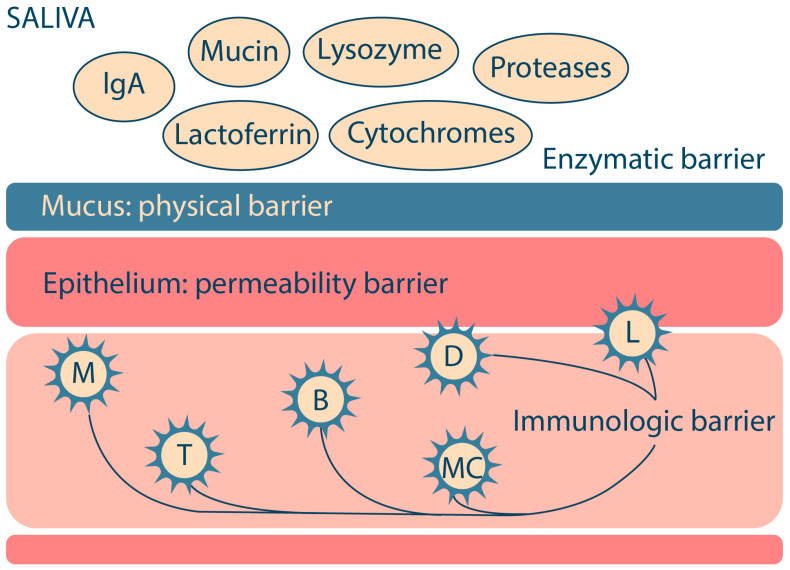
The major biological barriers of the oral cavity (M—macrophages; T—T-lymphocytes; B—B-lymphocytes; MC—mast cells; L—Langerhans cells; D—dendritic cells). Adapted from [18].

The main biological barriers of the oral cavity that protect against the entry of foreign substances into the body are the physical barrier (mucus), the permeability barrier (epithelium), the enzymatic barrier (proteases, cytochrome system, etc.) and the immunological barrier (immunocompetent cells) (Figure 1). These features of the oral cavity structure should be taken into account in the R&D of oromucosal dosage forms for effective drug absorption and high bioavailability [16,17].

### 2.1. Structure and Functions of the Oral Mucosa

**Figure 2:**
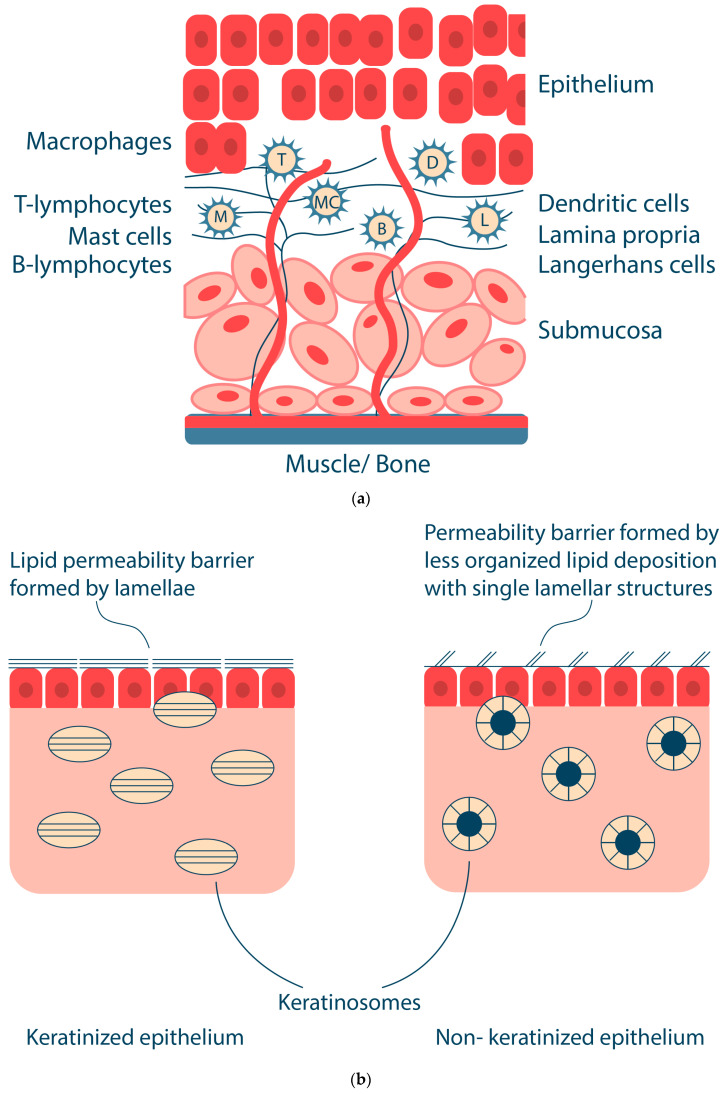
Oral mucosa: structure of mucosa (**a**) and keratinized and non-keratinized epithelia (**b**). Adapted from [18].

The oral mucosa has important protective functions: (i) protection of underlying tissues from mechanical damage, (ii) protection from microbial invasion, and (iii) a physical barrier to the entry of foreign substances, including drugs, into the body. The protective function of the oral mucosa is related to the physical structure of the epithelium, the presence of immunocompetent cells, including Langerhans cells and lymphocytes, and the secretion of various antimicrobial substances [19,20]. Oral fluids (e.g., saliva) contain a number of components, including free mucins, immunoglobulins, lysozyme, peroxidase, lactoferrin, proteases, etc., that are directed against foreign agents. The oral mucosa also has a sensory function due to the presence of various receptors sensitive to pain, temperature, etc. [18]. The key structures of the oral mucosa are the epithelium and the lamina propria (Figure 2a) [21].

The lamina propria is an amorphous connective tissue composed of cells, blood vessels, nerve elements, and supporting fibers. The lamina propria also contains macrophages, lymphocytes, mast cells, Langerhans cells, dendritic cells, etc. The oral mucosa in the buccal region and some other areas of the oral cavity have an additional structure, the submucosa, which consists of adipose and glandular connective tissue. The submucosa contains blood vessels, nerve fibers, and small salivary glands [21].

The epithelium of the oral cavity is the main protective barrier; it is a stratified squamous epithelium; the cells are connected by a variety of specialized transmembrane molecular complexes, including cell–cell junctions such as tight junctions, adherent junctions (desmosomes), and gap junctions [21]. These junctions provide selective permeability of the epithelium [22]. For example, tight junctions, a complex protein fibril-like structure, are responsible for regulating the transport of water, ions, solutes, and other molecules through paracellular pathways [23,24]. The turnover time of epithelial cells varies for different areas of the oral cavity; for example, buccal mucosa cells are renewed in 25 days [25].

The oral mucosal epithelium is divided into non-keratinized (epithelium of the sublingual and buccal mucosa) and keratinized (epithelium of the hard palate and gingiva), which is characterized by the presence or absence of a keratinized surface layer (Figure 2b). The cells of the keratinized epithelium of the oral cavity form several layers, namely stratum basale, stratum spinosum, stratum granulosum and stratum corneum [18,26]. The stratum spinosum is composed of keratinocytes that synthesize keratin (protein fibers). In the stratum granulosum, the process of keratinization begins, flattening the cells and creating keratinosomes that produce keratin and lipids in the upper layers of the epithelium. The stratum corneum consists of keratins embedded in a protein matrix with a lipid envelope consisting of ceramides, cholesterol and free fatty acids. This transforms keratinocytes into corneocytes, which are tightly bound together by adhesive structures called desmosomes and act as a physical barrier to foreign substances, including bacteria, as well as a water barrier [27,28]. The layers present in the non-keratinized epithelium include stratum basale, stratum filamentosum and stratum distendum [21]. The keratinosomes in the non-keratinized epithelium produce fewer lipids, which reduces the barrier function of the non-keratinized epithelium (including the buccal mucosa) and increases its permeability to bioactive substances [29]. Thus, the structure of the epithelium and the rate of renewal of epithelial cells is a barrier to drug absorption in the oral cavity. The physical and chemical connections between cells are due to intracellular protein-carbohydrate complexes produced by the epithelial cell within desmosomes and other connective complexes. The content and size of desmosomes are smaller in the non-keratinized epithelium. The non-keratinized epithelium has low enzymatic activity and higher permeability than the keratinized epithelium, which is used to deliver various drugs and vaccines through the oral cavity, mainly due to the different permeability of different parts of the oral cavity [30,31,32]. The difference in permeability between oral cavity regions decreases as follows: sublingual > buccal > palatal [33].

Such organelles as keratinosomes (lamellar or membrane-coated granules) play the main role in the formation of the permeability barrier [34]. The keratinosomes of keratinized epithelium are ovoid membrane-covered organelles, 0.25 μm long; those of non-keratinized epithelium are spherical membrane-covered vesicles, approximately 0.2 μm in size (Figure 2b) [35]. Keratinosomes contain acid hydrolases, sphingomyelin, phosphoglycerides, cholesterol, ceramides and other neutral lipids [36]. In keratinized oral epithelium, the permeability barrier is a neutral lipid or ceramide extruded from keratinosomes containing parallel lamellae [37]. In non-keratinized oral epithelium, the intercellular material also appears to be extruded from keratinosomes; however, it differs from the intercellular material in the keratinized epithelium in that it is amorphous and does not have a parallel lamellar structure [36]. The keratinized epithelium of the oral cavity contains mainly neutral lipids such as acylceramides and ceramides. In contrast, the non-keratinized epithelium of the buccal mucosa contains small amounts of ceramides and many polar lipids, such as cholesterol sulfate and glycosylceramides [38].

Thus, the permeability of the oral mucosa to bioactive molecules is possible due to the intercellular spaces of the apical epithelial layers. The presence of a lipid-containing keratinized layer on the apical surface reduces the permeability of the keratinized epithelium; therefore, sublingual and buccal administration are commonly used for transmucosal drug delivery.

### 2.2. Mucus and Mucins: Structure and Function

The apical surface of the epithelium is covered by a “glycocalyx” composed of various glycoproteins and mucins; the major glycoproteins are the mucins MUC5B and MUC7, as well as SIgA [39,40].

Mucus is a complex secretion synthesized by epithelial cells, consisting of water (95%) and mucins, as well as salts, lipids (including cholesterol, phospholipids, and fatty acids), and proteins (e.g., lysozyme, immunoglobulins, growth factors, etc.). However, the major component responsible for the viscous and elastic gel-like properties of mucus is the glycoprotein mucin [41]. Mucus provides protection from environmental factors through properties such as high viscosity and adhesiveness due to the presence of mucins. Mucus is also the first barrier through which drug molecules must diffuse to be absorbed into the systemic circulation and delivered to the desired tissues and target organs [42,43,44].

**Figure 3:**
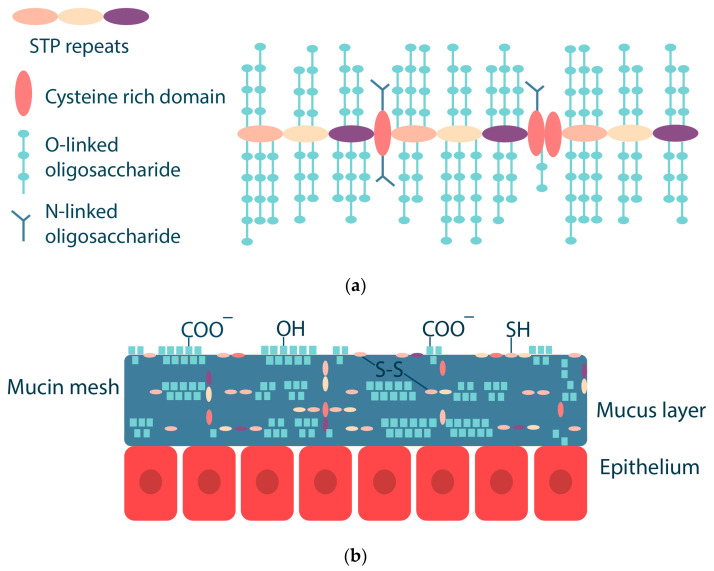
Schematic drawing of mucin consisting of different domains (**a**) and mucus layer structure (**b**). Adapted from [42].

Mucins are a key component of mucus, and they are glycoproteins with a size of 10–50 MDa. Mucins have a complex structure with different hydrophobic and hydrophilic domains [42,45]. Mucins consist of a protein core (20%) coated with an oligosaccharide shell (80%). Oligosaccharides consist of 5–15 monomeric units, which may include N-acetylgalactosamine, N-acetylglucosamine, fucose, galactose, sialic acid (N-acetylneuraminic acid), and others. Oligosaccharides are linked by O-glycosidic bonds to the hydroxyl-containing serine and threonine side chains of the protein core. The protein core of mucins has a molecular weight of about 200–500 kDa and consists of several domains: a central glycosylated domain rich in serine, threonine and proline (the so-called STP repeats) and domains with a small number of O-glycosylation and N-glycosylation sites rich in cysteine, which are capable of forming disulfide bonds (Figure 3) [42,46,47]. The conformation of mucin depends on pH and ionic strength; as pH decreases, there is a conformational change from an isotropic random helix at pH 7 to an anisotropic extended random helix at pH 2 [42]. The isoelectric point of mucins is in the range of pH 2–3 [48,49].

### 2.3. Mucoadhesion and Mucoadhesive Properties of Various Dosage Forms

Due to their complex structure, mucins are capable of forming hydrogen and ionic bonds as well as hydrophobic interactions; therefore, mucins can form complexes with other polymers and lipids. These properties are referred to as mucoadhesion [50]. Such mucoadhesive interactions are used to develop polymeric drug delivery systems with prolonged residence time on the mucosa due to intermolecular interactions of mucins with the polymeric shell of a drug nanocarrier, such as polyelectrolyte interactions of mucins with the polycation chitosan, hydrogen bonds with anionic and neutral polysaccharides containing hydroxyl, carboxyl or amine groups, and the formation of disulfide bridges with thiomers [51,52]. The following chemical bonds are responsible for mucoadhesion: strong covalent and ionic bonds, hydrogen bonds, and weak van der Waals bonds and hydrophobic interactions [53,54,55].

The following theories describe mucoadhesion [56,57,58]:

(i) Adsorption theory is based on the formation of chemical bonds of covalent and non-covalent nature (hydrogen bonds, electrostatic and hydrophobic interactions, and van der Waals forces) between the polymer and mucin;
(ii) Diffusion theory focuses on the entanglement of polymer chains with mucin macromolecules, resulting in the formation of a strong polymer network. Important characteristics of polymers that affect their diffusion properties are the flexibility of the polymer chain, the similarity of chemical structures and diffusion coefficients, and the contact time of the polymer with the mucosa;
(iii) The electron theory is based on the different electronic properties of polymers and mucins, which leads to electron transfer between the two surfaces; as a result, a doubly charged layer is formed at the mucus/polymer interface, allowing attraction and diffusion of the polymer with the mucin;
(iv) Fracture theory considers the strength of the adhesive bond between the polymer and the mucins as a function of the force required to detach the polymer layer from the mucosal surface;
(v) Mechanical theory is based on the process of adhesion of the polymer to the rough surface of the mucosa. The rough surface provides a larger contact area between the polymer and the mucosa and also provides increased viscoelastic and plastic energy dissipation during bond disruption, which is an important factor in mucoadhesion;
(vi) Wetting theory refers to the ability of the polymer to spread spontaneously over the mucosal surface; this ability is a necessary property for the occurrence of mucoadhesion.

It is important to note that mucoadhesion is a complex process that can be described by several of the above theories depending on the type of dosage form, e.g., solid (tablets, cryogel, patches, and polymeric micro- and nanoparticles), semi-solid (e.g., buccal gels for the treatment of periodontitis), or liquid (polymer-based oral drug solutions) [53].

**Figure 4:**
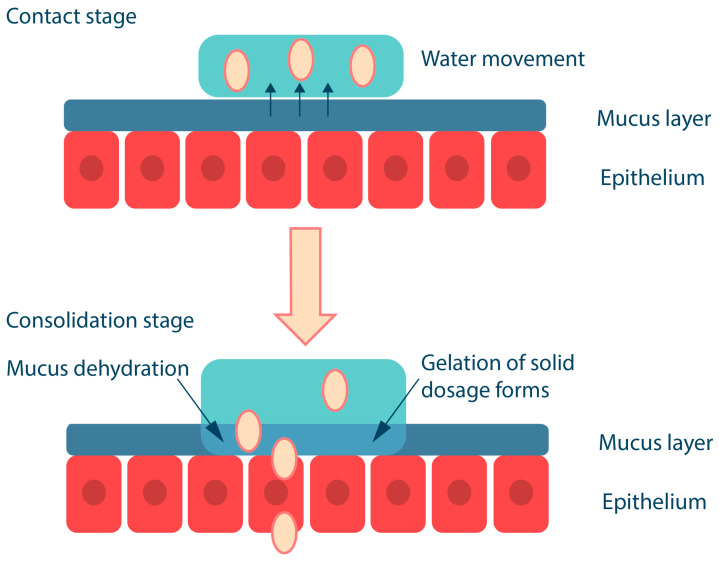
Mucoadhesion of solid polymer dosage forms. Adapted from [53].

Mucoadhesion of solid polymeric compositions occurs in two stages (Figure 4); the first is the contact stage, which is achieved by physical contact of the surfaces (e.g., by placing the solid dosage form on the buccal mucosa) or by deposition of the microparticles from the dispersion onto the mucosa (most commonly used for ocular or vaginal delivery, less commonly for buccal delivery). The second stage, referred to as the consolidation stage, involves the formation of a hydrogel layer in the presence of oral fluid; this releases and activates the mucoadhesive groups in the polymer, resulting in the formation of various chemical bonds and interactions between the polymer and mucin. The consolidation stage also involves interpenetration of macromolecular chains, whereby the polymer binds to mucus glycoproteins through secondary interactions, including entanglement [50,59]. In addition, upon contact with the mucus gel, the polymer, which has a high affinity for water and is capable of rapid polyelectrolyte gel formation, promotes the movement of a water molecule between the two gel layers; as a result, the polymer and mucus gel layers mix and solidify to an equilibrium state [60,61].

**Figure 5:**
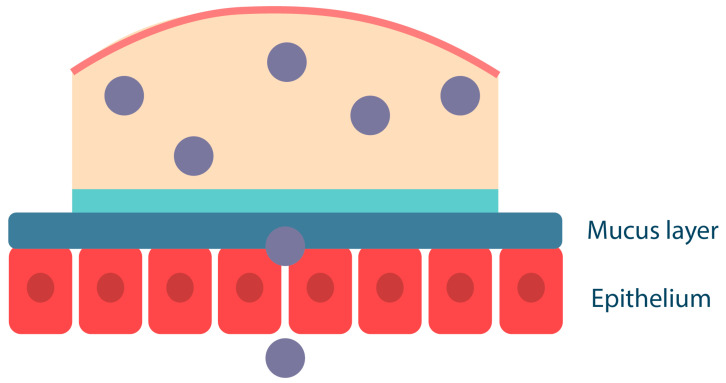
Mucoadhesion of semi-solid polymer dosage forms. Adapted from [53].

Semi-solid drug delivery systems, on the other hand, are already in the form of hydrated gels; macromolecular interaction and interpenetration occur at the interface between the polymer hydrogel and mucus without the contact stage (Figure 5). The mucoadhesion of hydrogels depends not only on the physicochemical properties of the polymers but also on their concentration (mucoadhesive gels with a high polymer content are retained on the mucosal surface for a long time) [62]. A semi-solid system should have optimal mobility to ensure maximum interaction with the mucosa and that it cannot be easily removed. However, semi-solid dosage forms have a significant disadvantage: they bind less strongly to the mucosa than solid systems and are, therefore, more easily removed from the mucosa by saliva as well as by ingestion. Such systems are suitable for topical administration when absorption into the systemic bloodstream is not required. They also do not provide unidirectional release, which is critical for drugs with high toxicity, such as corticosteroids, etc. [63].

**Figure 6:**
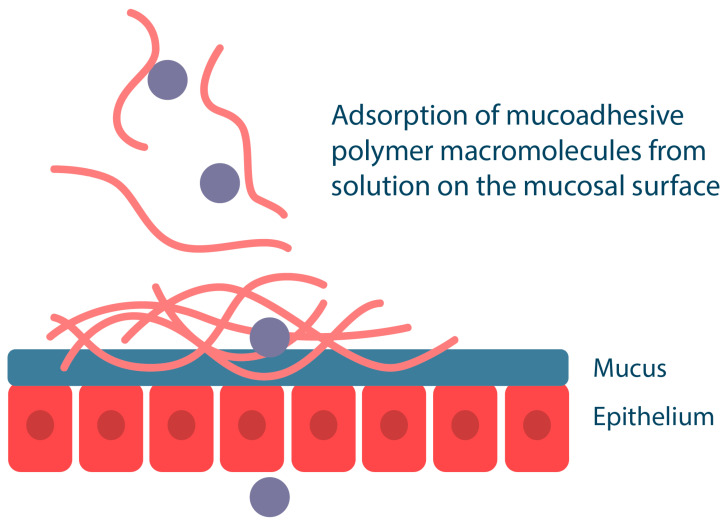
Mucoadhesion of liquid polymer dosage forms. Adapted from [53].

Mucoadhesive polymers can also be incorporated into liquid drug delivery systems, such as mouthwash solutions. In this case, mucoadhesion of the polymers to the mucosa occurs by deposition of the polymer component onto the mucosal surface, where the polymers compact and then bind to each other and to mucin by forming various chemical bonds and by entangling macromolecular chains (Figure 6). For example, the cationic polymer chitosan in dilute solutions can bind to buccal cells in vitro and persist for more than two hours in vivo [64,65]. The disadvantage of liquid drug delivery systems is their high mobility, which makes them easy to remove from the mucosal surface compared to semi-solid gels or solid tablets and patches. It should be noted that such systems do not provide unidirectional drug release; liquid polymeric systems are suitable for local drug delivery for extensive oral lesions, such as infectious diseases.

### 2.4. Overview of Mucoadhesion Assays

In the pharmaceutical development of oromucosal delivery systems, there is a need for reliable methods to determine mucoadhesion and predict the in vivo interaction of the delivery system with the mucosa in order to evaluate the prospective clinical use of the formulations. Methods for evaluating the mucoadhesion of oromucosal drug delivery systems can be categorized into in vivo, ex vivo, and in vitro assays. In vivo and ex vivo methods (e.g., rat intestinal fragment model) are the most accurate, but their use is limited for ethical reasons [66,67]. At the same time, in vitro tests are express and convenient, do not require the use of animals, and allow reliable prediction of the in vivo mucoadhesive potential of novel dosage forms. The main disadvantage of these tests is that they do not perfectly reflect in vivo processes [50].

The most commonly used in vitro tests to evaluate the mucoadhesion of biopolymer systems are:

(1) Tensile strength (mechanical method), which is based on measuring the detachment force of the test material from the mucosal surface. This method uses an instrument such as a texture analyzer and can be used to study solid and semi-solid formulations. A variation of this method is the shear test, which measures the force required to detach mucoadhesive samples layer by layer (this test is specific to mucoadhesive patches [50]);
(2) The method of dynamic and electrophoretic light scattering is based on the measurement of hydrodynamic diameters and ζ-potentials of mucin particles before and after interaction with liquid mucoadhesive systems [68]. For example, the study [69] has shown that the determination of the ζ-potential of chitosan nanoparticles in the presence of mucin is a fast and convenient technique to study the mucoadhesive properties of chitosan nanoparticles cross-linked with tripolyphosphate. The interaction of NH_3_^+^ groups of chitosan with ionized COO^−^ groups of sialic acid of mucin resulted in a significant increase in the hydrodynamic size of the obtained particles and a decrease in their ζ-potential;
(3) Spectrophotometric methods, namely colorimetric and turbidimetric methods. The colorimetric method is based on the determination of the amount of mucin adsorbed on the surface of mucoadhesive biopolymer particles. The biopolymer system is incubated with mucin solution and separated. The non-adsorbed mucin is then treated with periodic acid to oxidize the hydroxyl groups to aldehyde groups and quantified using Schiff’s reagent [70]. This method is convenient and reproducible, correlates well with in vivo conditions, and has been widely used to determine the mucoadhesion properties of chitosan-based biopolymer delivery systems [69]. The turbidimetric titration method can also be used to study the mucoadhesive properties of polymers. In turbidimetric titration, polymers with high affinity to mucin easily form aggregates, which changes the turbidity of the system. The method can also be used to estimate the degree of polymer-mucin complexation [71];
(4) Rheological method. The interaction of polymer gel with mucins can be evaluated by increasing the viscosity of the system through rheological synergism due to the rearrangement of macromolecules [51]. This behavior is characteristic of anionic macromolecules that possess both many hydrogen-bonding functional groups and a branched structure (e.g., carrageenans) [72]. However, polycationic molecules (e.g., chitosan) that form particles upon interaction with mucins are then characterized by a decrease in the viscosity of the system [69].

Less precise methods for studying mucoadhesion are atomic force microscopy (namely, the colloidal probe technique, which is used to study mucoadhesive properties by examining the change in surface roughness due to binding between the mucoadhesive polymer nanoparticle and the mucus layer), isothermal titration calorimetry to determine the thermodynamic parameters of the interaction between mucins and polymers, and the surface plasmon resonance method [73,74].

## 3. Drug Absorption in the Oral Cavity: Transcellular and Paracellular Transport

**Figure 7:**
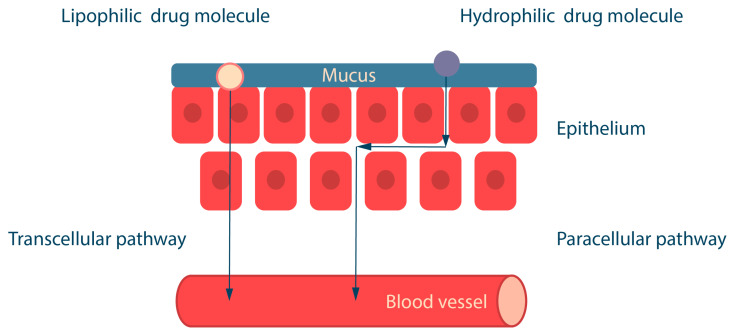
Drug absorption pathways across the epithelium. Adapted from [16].

The design of polymeric buccal drug delivery systems should take into account the physicochemical properties of the drugs, including their solubility, octanol/water partition coefficient (logP), and degree of ionization at different pH (pKa) [75,76,77]. The epithelium consists of both hydrophobic sites (lipophilic epithelial cell membranes) and hydrophilic sites (intercellular space) [78]. Depending on their properties, drugs can be absorbed in the oral cavity by two routes: (i) hydrophobic compounds are absorbed by the transcellular route through cell membranes, and (ii) hydrophilic compounds are absorbed by the paracellular route through the intercellular space by passive diffusion (Figure 7) [79,80].

Lipophilic substances usually contain a benzene ring, a steroid nucleus, a carbohydrate chain or halogens in their structure. The ability of lipophilic molecules to cross the lipid barrier depends on their lipid solubility, which is determined by logP [81]. In general, small and lipophilic molecules with logP around 1.6–3.3 are well absorbed across the oral mucosa, whereas drugs with higher logP values are poorly absorbed due to their low water solubility [16]. Water-soluble substances have chemical groups in their structure, such as alcohol (-OH), amide (-CONH_2_) and carboxyl (-COOH), which are capable of forming conjugates with glucuronic and sulfate moieties [82].

The degree of ionization of a drug at different pH values depends on the pKa value and affects the solubility of substances and their route of absorption across the mucosa. Ionized molecules are water-soluble (paracellular route), and non-ionized molecules are lipid-soluble (transcellular route). At physiological pH 7.4, non-ionized molecules are lipid soluble and diffuse across the epithelium. Acids are less ionized at acidic pH, and bases are less ionized at alkaline pH. The pH of the oral cavity is approximately 7. Therefore, the drug substance for buccal delivery should be ionized at the site of absorption for better epithelium permeability [83]. Permeability is reduced for drugs that are in an ionized state at the pH of the oral cavity (around 6), so drug permeability can be improved by methods that increase the non-ionized fraction of the drug. For example, the use of aprotic solvents such as dimethylacetamide, dimethylacetamide, decyl methyl sulfoxide, and dimethylsulfoxide [84,85,86].

Other effective strategies to improve bioavailability include the use of nanoparticles (see Section 6) and the use of various excipients such as permeability enhancers and protease inhibitors [16,87,88]. Protease inhibitors (e.g., aprotinin, amastatin, puromycin, boroleucin, etc.) can overcome the enzymatic barrier present in saliva and mucus, thus improving buccal drug delivery by preventing their enzymatic degradation [16,89]. Protease inhibitors can also alter the pH in the oral cavity, change the conformation of the peptide or protein, and bind to the active domains of the enzyme, reducing the number of sites available for enzymatic degradation. As a result, enzymatic activity is reduced, the stability of the drug in the oral environment is increased, and buccal bioavailability is improved [90,91].

Permeability enhancers can increase drug absorption by (i) increasing drug distribution, (ii) interacting with epithelial cell protein domains, (iii) extracting intercellular lipids, creating a space for molecules to pass between adjacent cells, and (iv) increasing the solubility of drugs in the delivery system [76,91]. Permeability enhancers also reduce the viscosity and elasticity of mucus, resulting in improved diffusion of macromolecules. The following types of permeability enhancers are commonly used to improve buccal delivery: surfactants (sodium lauryl sulfate, lysophosphatidylcholine, sodium dioctylsulfosuccinate, polysorbate 80, etc.), fatty acids (sucrose palmitate, oleic acid, lauric acid, etc.) and mucoadhesive biopolymers such as chitosan, sodium carboxymethyl cellulose (CMC), etc. [92,93,94,95,96].

## 4. Mucoadhesive Biopolymers

The ability of biopolymers to adhere to the mucosal surface has been successfully exploited in the R&D of innovative dosage forms for buccal or oromucosal drug delivery because it allows prolonging the residence time of the drug at the target site. Typically, the following physicochemical properties are considered to select a suitable biopolymer with the desired mucoadhesion [51,97]:

(1) The molecular weight of the polymer should generally be in the range of 10 to 4000 kDa, which allows polymers to hydrate easily, releasing active groups for interaction with mucin and, at the same time, forming strong gels; polymers with a lower MW pass easily into a dissolved state, while those with a higher MW are poorly hydrated; the length of the polymer chain should be optimal on the one hand to promote interpenetration of macromolecules and on the other hand not to hinder diffusion;
(2) Molecular conformation: the helical conformation of some biopolymers, such as dextrans, protects the chemical groups responsible for mucoadhesion, in contrast to linear biopolymers, whose conformation provides accessibility for chemical interactions with mucin;
(3) The flexibility of the polymer chains allows interpenetration and adhesion of the polymer molecules with the mucins; the optimal degree of cross-linking of the polymers has a positive effect on gel formation and mucoadhesion and thus provides a controlled release of the drug;
(4) The mucoadhesive properties of polymers containing ionizable groups are affected by pH, e.g., for high mucoadhesion of polymers with a large number of carboxylate groups, the groups must be in non-ionized form (i.e., at pH below pKa). pH also affects positively charged polymers, e.g., the protonated amino groups of chitosan form ionic bonds with mucins, enhancing mucoadhesion. pH can also alter the conformation of macromolecules.

Various biopolymers, especially polysaccharides and their derivatives such as chitosan, hyaluronic acid, alginate, carrageenan, and cellulose derivatives, etc., have been used as mucoadhesive carriers in the development of drug delivery systems with enhanced mucoadhesiveness [98,99,100]. Biopolymers often have intrinsic pharmacological activities, including antimicrobial, antifungal, antitumor, immunomodulatory, and antiviral effects, as well as wound healing (controlling inflammatory responses, stimulating cell growth and migration, stimulating angiogenesis, promoting re-epithelialization, and reducing scarring), and others [101,102,103,104,105].

### 4.1. Cationic Biopolymers

The cationic polysaccharide chitosan is a well-known mucoadhesive biopolymer [106,107]. The chitosan macromolecule consists of glucosamine and N-acetylglucosamine residues; chitosans have a wide range of molecular weights (from oligomers with MW of about 2 kDa to polymers of 50 kDa and higher) and degrees of deacetylation (40–98%). Chitosan is widely used as the main component of mucoadhesive systems for buccal and oromucosal drug delivery [108]. At physiological pH, the mucoadhesion of chitosan is mainly due to ionic bonds between partially protonated amino groups of chitosan and negatively charged groups of mucins, as well as hydrogen bonds and hydrophobic interactions [109]. To improve mucoadhesion, the chitosan is modified by the addition of substituents with high cationic density. For example, N-trimethylchitosan, a partially quaternized derivative of chitosan, shows improved mucoadhesion with an increasing degree of quaternization compared to native chitosan [106,110]. Diethylaminoethyl chitosan is also characterized by improved mucoadhesive properties depending on the degree of substitution and the degree of quaternization [111,112,113]. Thiolated chitosan shows improved mucoadhesion due to thiol groups forming disulfide bridges with cysteine groups in the mucin [114,115]. In addition to mucoadhesion, the second important property of chitosan and chitosan derivatives is the ability to enhance the absorption of hydrophilic drugs across the mucosal epithelium. Protonated chitosan molecules at pH 6.5 can increase paracellular permeability by opening tight junctions [95,106].

### 4.2. Anionic Biopolymers

Anionic polymers have a large number of carboxyl and sulfate functional groups, resulting in a negative charge at pH values above pKa. Anionic polymers have excellent mucoadhesive properties due to the formation of strong hydrogen bonds with mucins. Common polyanionic polymers include alginate, hyaluronic acid, pectin, CMC, and sulfated biopolymers such as carrageenans, fucoidans, etc. [51,116,117,118]. For example, Szekalska et al. [101] developed a polymeric film for buccal delivery of the antifungal drug posaconazole. They used alginate as a mucoadhesive biopolymer as well as its oligosaccharide oligomer consisting mainly of mannuronic acid. The film containing 1% alginate and 1% oligomer was the most promising; this formulation was characterized by optimal mucoadhesive and swelling properties as well as ex vivo and in vivo drug residence time. The resulting film provided prolonged drug release for up to 5 h. Furthermore, the presence of the oligomer in the film composition significantly reduced the growth of *Candida* sp.

Pamlény et al. [119] prepared buccal mucoadhesive films with cetirizine based on the biopolymers sodium alginate and hydroxypropyl methylcellulose (HPMC) in 1:1 and 2:1 ratios. It was shown that the in vitro mucoadhesion of the obtained films was extremely high; however, it decreased in the presence of cetirizine and the plasticizer glycerol, apparently due to the fact that these compounds are able to form hydrogen bonds with the mucoadhesive active groups of the polymers. In general, the strength of mucoadhesion could be controlled by increasing the concentration of polymers in the film-forming solution. Despite the fact that the mucoadhesive properties of HPMC are higher than those of alginate, varying the ratio of polymers did not significantly affect the mucoadhesion of the films. At the same time, the amount of alginate influenced the drug release: in films with an alginate:HPMC ratio of 1:1, cetirizine was released in 5–10 min, whereas with a ratio of 2:1, 60% of the cetirizine was released gradually within 20 min, with the remaining 40% of the drug released within 120 min.

Abo-shady et al. [102] used hyaluronic acid to prepare a mucoadhesive buccal film as a novel delivery system to overcome the disadvantages of traditional dosage forms (mouthwashes and gels) used to treat oral aphthous ulcers. Polyvinyl alcohol and hydroxyethyl cellulose were also used as excipients to form the film. A film based on hyaluronic acid and hydroxyethyl cellulose in a ratio of 1:15 showed excellent results (good mechanical properties, high mucoadhesion and controlled release of hyaluronic acid as a bioactive wound healing component). Clinical studies demonstrated the resistance of the developed film to saliva and food erosion and a significant therapeutic effect in the treatment of aphthous ulcers compared to a commercial gel.

Pectin and gums are also promising biopolymers for the development of mucoadhesive drug delivery systems for oral cavity applications. For example, Prezotti et al. [120] developed buccal films based on biopolymers of gellan gum and pectin. The mass ratios of gellan gum:pectin (4:1; 1:1; 1:4) and polymer concentrations (3% or 4%) were varied, and glycerin (1 or 2%) was also used as a plasticizer. The resulting films had high mechanical strength and high mucoadhesion. It was shown that increasing the gellan gum content improved both the mechanical strength and mucoadhesion of the films. The developed films provided sustained release of curcumin for up to 12 h and were also stable in artificial saliva for up to 24 h.

Among the sulfated polysaccharides, carrageenans and fucoidans are the most widely used for biomedical applications [121]. The main types of carrageenans used in the pharmaceutical development of mucoadhesive formulations are κ-, ι- and λ-carrageenan. The main differences in these carrageenans are due to the number and position of the ester sulfate groups within the galactose repeating units [51,122,123]. Carrageenans, along with alginate, hypromellose, and cellulose, are alternatives to animal gelatin in the manufacture of hard capsules [124]. An important property of carrageenans is their gelling ability, which is highly dependent on the degree of sulfation; due to the gelling properties, various lyophilized structures (i.e., lyophilized wafers, sponges, and cryogels) can be prepared based on carrageenans. For example, Kianfar et al. [125] developed a technology of lyophilized mucoadhesive wafers containing paracetamol (water-soluble drug) or ibuprofen (water-insoluble drug) for potential oral applications. The lyophilized wafers were prepared by lyophilizing gels containing the natural polysaccharide κ-carrageenan and pluronic acid. It was shown that the crystalline forms of the drugs were converted to amorphous form during gel formation and lyophilization, with the biopolymer matrix of the lyophilized wafers retaining both drugs in stable amorphous form during storage for 6 years. The developed lyophilized wafers exhibited ideal mechanical and mucoadhesive properties at a carrageenan:pluronic acid component ratio of 1:2. Both drugs were released gradually and prolonged in a saliva-simulating fluid within 2 h. The developed oromucosal dosage form is well established for both topical and buccal application.

Elbanna et al. [126] obtained mucoadhesive composite sponges based on κ-carrageenan and xanthan biopolymers for buccal delivery of tetrahydrocurcumin for the prevention and treatment of oral cancer. The developed sponges had good mucoadhesion properties (0.24 N) and prolonged drug release for 4 days. The chemopreventive activity of the sponges was evaluated in a model of oral precancerous lesions induced by the carcinogen 7,12-dimethylbenz(a)anthracene. As a result, a significant regression in the degree of dysplasia was shown, with a decrease in the mean area of cyclin D1 immunoexpression after 25 days of daily application of the sponge (12%) compared to the control (40%).

Fucoidans are a heterogeneous group of polysaccharides negatively charged by sulfate ester groups; fucoidans have various bioactivities, including antitumor and antiviral. Fucoidans are characterized by a wide range of physicochemical properties depending on their molecular weight, monomer composition, degree of sulfation, degree of branching, etc., which should be taken into account when developing pharmaceutical formulations [103,127]. An important aspect of the use of fucoidan-based topical drug delivery systems with improved properties (e.g., synergism and potentiation of pharmacological effect due to the biological activity of fucoidan, etc.) is the treatment of acute and chronic wounds, including a number of lesions of the oral mucosa [128].

For example, Zheng et al. [129] developed oromucosal patches based on chemically cross-linked chitosan/fucoidan hydrogels loaded with triamcinolone acetonide for the treatment of inflammatory oral diseases. It was shown that the incorporation of fucoidan into chitosan hydrogels significantly improved their swelling, mechanical strength and mucoadhesion. Triamcinolone acetonide showed prolonged release from the developed patches, which effectively suppressed the inflammatory response and promoted the formation of well-organized collagen fibers. In another study, Dou et al. [130] developed hydrogel patches based on fucoidan and calcium alginate to load probiotics for the treatment of oral ulcers. The developed patches showed excellent mucoadhesion to moist tissues and also had suitable swelling and mechanical properties, prolonged release of probiotics and storage stability. The bioactive hydrogel patches showed a high rate of ulcer healing in vivo by enhancing cell migration, inducing the formation of epithelium and well-organized collagen fibers, and facilitating neovascularization.

### 4.3. Non-Ionic Biopolymers

Non-ionic polymers such as starch, cellulose and cellulose derivatives, such as HPMC, methylcellulose, etc., have also been used to develop mucoadhesive drug delivery systems [51]. For example, Meher et al. [131] developed a mucoadhesive polymer film based on cellulose derivatives (methylcellulose and HPMC) and polymethacrylate (Eudragit RSPO) for the buccal delivery of carvedilol. It was shown that an equal ratio of HPMC K4M (4000 cP) and HPMC K15M (15,000 cP) in combination with methylcellulose, Eudragit RSPO and Carbopol 940 provided the best mucoadhesive properties. In this case, HPMC was a key component in controlling the release of carvedilol. The mucoadhesive film demonstrated 88% in vitro drug release in 12 h and 80% ex vivo penetration of carvedilol through goat buccal mucosa in 12 h.

However, non-ionic polymers are characterized by poorer mucoadhesiveness compared to polyelectrolytes [50,51]; therefore, their chemical modification with adhesive functional groups is used to improve mucoadhesiveness [132]. For example, Kali et al. [133] prepared cellulose modified with 2-mercaptosuccinic acid by reacting cellulose with S-acetylmercaptosuccinic anhydride. The synthesized conjugate contained 215 μmol/g thiol groups and 84 μmol/g disulfide bonds. As a result of thiolation, the mucoadhesion of the resulting derivative increased 9.6-fold. In the presence of thiolated cellulose, permeability across the mucosa was enhanced; e.g., the apparent permeability coefficient (Papp) of the model compound increased 2.2-fold with the addition of 0.5% cellulose mercaptosuccinate (Caco-2 cell model). The penetration of enoxaparin through the intestinal mucosa of rats increased 2.4-fold in a 0.5% cellulose mercaptosuccinate solution compared to a solution of the drug in buffer. Similarly, in in vivo experiments in a rat model, the oral bioavailability of enoxaparin was 12.5 times higher in the cellulose mercaptosuccinate solution than in the aqueous solution. This strategy may also be successful for transbuccal drug delivery.

Starch is a mixture of the polysaccharides amylose and amylopectin, which consist of monomeric alpha-glucose residues linked by glycosidic bonds [51]. The starches used are derived from potatoes, corn (maize), wheat, rice, and cassava roots. Native starches vary in viscosity, clarity, and syneresis, but these properties can be adjusted by modifying the native starch. Typically, starches are modified by physical, chemical, or enzymatic means [134]. The mucoadhesive properties of native starches are well known (e.g., maize starch and waxy maize starch), as are those of pregelatinized waxy maize starch [135]. Starch gels also actively absorb water, resulting in mucosal dehydration, which enhances drug permeation through paracellular tight junctions [136].

Mulhbacher et al. [137] investigated the mucoadhesive properties of acetate (Ac), aminoethyl (AE) and carboxymethyl (CM) derivatives of cross-linked high amylose starch (HAS) for further use in buccal mucosal delivery. The ionic derivatives AE-HAS and CM-HAS were shown to have higher mucoadhesive properties than the neutral HAS and Ac-HAS. In this case, swelling and mucoadhesion depended on the type of ionic substituent and pH. Negatively charged derivatives (CM-HAS) showed better mucoadhesion at neutral pH and can, therefore, be used as polymer matrices for buccal drug delivery. On the other hand, positively charged derivatives (AE-HAS) showed better mucoadhesion at acidic pH and can be used, for example, for vaginal delivery of bioactive substances.

As an alternative to animal-derived chitosan, cationic starch derivatives can be used. For example, Jelkmann et al. [138] prepared cationic starch derivatives by reductive amination. The synthesis was carried out in two steps. First, oxidative ring opening of the polysaccharide structure and oxidation of vicinal diols with the formation of aldehyde groups were performed in the presence of NaIO_4_. Second, primary amines were introduced by reductive amination with ammonia in the presence of an ethanol solution of sodium cyanoborohydride. Aminated starch with a high degree of substitution (514 µmol/g) showed a 9.5-fold prolonged residence time on the mucosa and a 2.7-fold higher adhesion to the mucosal tissue compared to chitosan. In addition, the starch derivatives exhibited even less cytotoxicity than chitosan.

### 4.4. Polyampholyte Biopolymers

Polyampholytes are polymers containing both anionic and cationic groups in their structure, which makes them characterized by the presence of an isoelectric point (pH at which the macromolecules carry no net electrical charge) [139]. The properties of the polyampholytes can be adjusted by varying the pH because, at pH below or above the isoelectric point, the polyampholytes are positively or negatively charged, respectively. Examples of polyampholytic biopolymers are proteins containing amino acid residues of both acidic (e.g., aspartic and glutamic acid) and basic (e.g., lysine and arginine) nature [140]. Known pharmaceutical excipients of a protein nature, such as collagen-derived gelatin, have poor mucoadhesive properties comparable to those of non-ionic polymers [141]. In general, the mucoadhesion of polyampholytes can be improved by chemical modification. For example, Wang et al. [142] introduced amino groups into gelatin by reaction with ethylenediamine in the presence of 1-ethyl-3-(3-dimethylaminopropyl)-carbodiimide hydrochloride. The derived aminated gelatin showed improved mucoadhesion compared to pure gelatin in in vitro (an isolated rat stomach model) and in vivo experiments in a male Wistar rat model [143].

### 4.5. Thiolated Biopolymers (Thiomers)

Thiolated biopolymers have high mucoadhesive properties due to the formation of strong covalent disulfide bonds with cysteine-rich domains of mucus glycoproteins through the process of thiol-disulfide exchange reactions [144]. In general, thiolated biopolymers provide higher mucoadhesion and drug residence time compared to native polymers, thereby increasing drug bioavailability. Thiolated biopolymers also inhibit some enzymes, thereby increasing drug stability, as well as providing controlled drug release and increasing cell permeability [145,146]. The most commonly used thiolating agents are thioglycolic acid, thiourea, 4-aminothiophenol, L-cysteine and Trout’s reagent (2-iminothiolane).

Due to their properties, thiolated biopolymers are actively used for the development of oromucosal dosage forms. For example, thiolated chitosan has been used for buccal insulin delivery [147,148,149]. For example, Rahbarian et al. [147] developed polyelectrolyte particles for buccal insulin delivery based on quaternized chitosan, triethylchitosan thiolated with L-cysteine (19% degree of quaternization and 165 μmol thiol group/g of polymer). The resulting particles had suitable physicochemical properties (hydrodynamic size 127 nm and ζ-potential 24.6 mV) and a high encapsulation efficiency of about 98%. The in vitro insulin release was 98% within 8 h (PBS, pH = 7.4); the ex vivo permeability of encapsulated insulin across rabbit buccal mucosa reached 96% in 8 h.

Özbaş et al. [150] prepared a buccal mucoadhesive patch with triamcinolone acetonide based on alginate and pectin thiolated with L-cysteine. SH-modified patches were characterized by 1.5-fold higher swelling and 3-fold enhanced ex vivo mucoadhesion to bovine buccal mucosa compared to unmodified patches. Both patches showed prolonged drug release for 50 h. Özkahraman et al. [151] obtained oromucosal patches based on pectin and thiolated κ-carrageenan for the treatment of oral fungal infections. For this purpose, κ-carrageenan was grafted with acrylic acid (the grafting percentage was 85%) and then modified with L-cysteine (sulfur content was 68%) and 3-mercaptopropionic acid (sulfur content was 78%). Swelling studies showed that the degree of swelling of the hydrogel patches and their mucoadhesion strength increased with increasing number of thiol groups in the system; the highest swelling and mucoadhesion were characterized by the patches composed of pectin and MPA-κ-carrageenan in the ratio of 1:9. The resulting patches showed prolonged drug release for 8 h (simulated oral fluid, pH 6.8) and high efficacy against *Aspergillus fumigatus* and *Aspergillus flavus*.

However, the disadvantage of thiomers is that the disulfide bond is reversible, so it is relevant to look for other molecules to modify biopolymers to improve their mucoadhesion. Currently, polymers with catechol moieties are being considered as mucoadhesion enhancers, for example, marine mussel adhesive proteins, which exhibit superior adhesion properties due to the presence of dopamine [152,153,154].

### 4.6. Catechol-Based Adhesive Biopolymers as Products of Mussel-Inspired Chemistry

**Figure 8:**
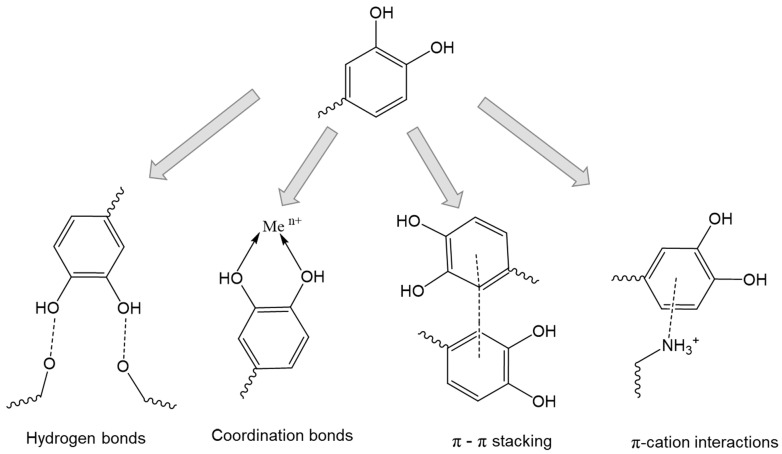
Chemical interactions of catechol-containing polymers. Adapted from [156].

Mussel-inspired chemistry is a new, effective strategy to modify biopolymers to improve their mucoadhesion, oxidation resistance, chelating and coordinating abilities, and antibacterial properties [152,155]. In fact, mussels secrete a special mucus whose ultra-adhesive properties are due to the catechol moiety of dopamine. The structures containing catechol moieties are responsible for the extremely strong adhesive ability of various marine animals (e.g., mussels), even in aqueous environments in the presence of competing water molecules, which can be used to modify biopolymers to improve their mucoadhesion [156,157,158,159,160,161,162]. Catechol, the ortho isomer of 1,2-dihydroxybenzene, is able to bind to mucins by forming hydrogen bonds due to the presence of hydroxyl groups in the ortho position [160]. An important advantage of catechol is its availability for conjugation, including with bioactive molecules; catechol is readily oxidized to the quinone form [163], which is highly reactive toward various functional groups, including thiol and amino groups via Michael addition or Schiff base reactions. Catechol moieties can be used for conjugation with metals and metal oxides (i.e., Ag^+^, Fe^3+^, etc.) via coordination bonds [164,165,166,167]. Also, a unique combination of hydroxyl and phenolic groups can promote π-π stacking and π-cation interactions (Figure 8) [156].

Dopamine, hydrocaffeic acid (3,4-dihydroxyhydrocinnamic acid) and tannic acid are key reagents in mussel-inspired chemistry [12,71,155]. Dopamine and hydrocaffeic acid, which contain a catechol functional group, are responsible for their adhesive properties [168,169]. Tannic acid is composed of five pyrogallol and five catechol groups and is capable of a variety of interactions, such as hydrogen bonding, ionic bonding, coordination, and hydrophobic interaction with other materials. As a result, it strongly adheres to various substrates, including mucous membranes [169,170].

The mussel-inspired chemistry strategy has been successfully applied to the modification of biopolymers to improve their mucoadhesive properties and to the pharmaceutical development of buccal drug delivery systems with unique properties. For example, Lee et al. [171] developed a thermosensitive cross-linked gel based on hyaluronic acid and poly(ethylene oxide)-poly(propylene oxide)-poly(ethylene oxide) copolymer (PEO-PPO-PEO-PEO, Pluronic). To improve the mucoadhesion of the resulting system, hyaluronic acid was modified with dopamine by carbodiimide cross-linking between the carboxyl groups of hyaluronic acid and the amino groups of dopamine in the presence of 1-Ethyl-3-(3-dimethylaminopropyl)carbodiimide catalyst (degrees of substitution with dopamine were 24 and 47%). The terminal hydroxyl groups of pluronic acid were modified with cysteamine in the presence of p-nitrophenyl chloroformate and triethylamine to form terminal thiol groups (degree of thiolation was 94%). Hyaluronan/Pluronic composite hydrogels were then synthesized by homogeneously mixing different amounts of hyaluronan- dopamine and Pluronic-SH using the Michael-type catechol thiol addition reaction. The resulting cross-linked hydrogels exhibited significantly improved tissue adhesion properties; the adhesion strength of the hyaluronan/Pluronic hydrogel was 414% higher than that of the physical mixture of hyaluronic acid and Pluronic.

Kim et al. [71] synthesized chitosan modified with hydrocaffeic acid (degree of substitution ranged from 7% to 20%). Pure chitosan is known to rapidly contact mucins through electrostatic interactions. The authors hypothesized that the presence of catechol moieties in chitosan would provide a cooperative two-step mechanism at the consolidation stage through the formation of additional hydrogen and covalent bonds. The study of adhesion by surface plasmon resonance showed that the mucoadhesion of catechol-modified chitosan was 3–4 times higher than that of the original chitosan, and there was a positive correlation between the enhancement of mucoadhesion and the increase in the number of catechol moieties in the macromolecule. Moreover, the rapid association of catechol-chitosan with mucin, followed by the formation of a covalent bond with cysteine thiolate, provided ultra-long mucoadhesion (about 10 h) to the intestinal mucosa (unmodified chitosan was removed within 3 h).

Xu et al. [12] developed a cross-linked gel with enhanced mucoadhesion based on chitosan modified with hydrocaffeic acid (degrees of substitution were 9% and 19%). High degrees of substitution with catechol moieties in chitosan significantly enhanced in vitro mucoadhesion to porcine mucosa for up to 6 h compared to pure chitosan (1.5 h). Buccal patches based on the obtained cross-linked hydrogel in in vivo experiments (rabbit buccal mucosa model) provided a concentration of the model drug lidocaine in rabbit serum at a therapeutic concentration of about 1 ng/mL. The resulting systems had no local toxic or inflammatory effects on rabbit oral cavity tissues.

In conclusion, the use of catechol-modified biopolymers is currently a simple and reliable approach for the development of highly effective mucoadhesive systems for oromucosal drug delivery.

The key properties of mucoadhesive biopolymers that may be useful in the pharmaceutical development of oromucosal delivery systems are summarized in Table 1.

## 5. Dosage Forms for Oromucosal Application

Oromucosal dosage forms can be topical and systemic (transmucosal systems that ensure absorption of the drug into the systemic bloodstream). Topical delivery is more suitable for the treatment of various oral mucosal pathologies caused by inflammatory, autoimmune and infectious factors. In turn, dosage forms for transmucosal delivery should provide high oral bioavailability (adequate rate of absorption of drugs into the bloodstream at a given therapeutic level) [172,173]. The buccal mucosa is poorly permeable to drugs and does not provide high bioavailability compared to sublingual administration, but the sublingual mucosa is irregular and mobile and constantly washed with saliva, making controlled transmucosal drug delivery problematic (except for fast-acting formulations, e.g., angina sprays, etc.). Therefore, the buccal mucosa is the preferred site in the oral cavity for the attachment of a polymeric system for programmable and controlled transmucosal drug delivery [174]. Nowadays, several oramucosal mucoadhesive systems have been developed, such as solid dosage forms (patches, tablets) as well as gels, polymer solutions, etc. [6,175].

One of the first oromucosal dosage forms (1959) is Orabase^®^, a gelatin, pectin, and CMC film vehicle for drug delivery to the oral mucosa [176]. The concept of oromucosal drug delivery systems has evolved from polymer solutions, gels, and sprays (e.g., Corsodyl^®^, chlorhexidine bigluconate gel) to buccal tablets (e.g., Corlan^®^, hydrocortisone buccal tablets) and patches (e.g., Belbuca^®^, buprenorphine buccal film), including multilayer patches. For example, Breakyl^®^, the first fentanyl formulation in a bilayer mucoadhesive buccal film, was launched in 2014. To improve the efficacy and safety of fentanyl, the drug was incorporated into the mucoadhesive layer and the top protective layer provided unidirectional drug release. The oromucosal films as a dosage form were included in the European Pharmacopoeia (monograph “Oromucosal preparations”) in 2012 [177,178,179].

The general requirements for oromucosal forms are (i) improved residence time on the oral mucosa and stability in a humid environment, (ii) a modified release profile (programmed and controlled release), including unidirectional release of the drug to prevent entry into the gastrointestinal tract. In addition, a specific requirement for transbuccal dosage forms is improved absorption of the drug through mucosal cells into the systemic bloodstream. An important requirement for topical dosage forms is to ensure that the drug does not enter the gastrointestinal tract with saliva or food. In general, solid dosage forms should be small and flexible to be convenient for patients [175].

Solid dosage forms for oromucosal use are best suited for all major requirements. These systems are of several types, such as (1) monolayer systems with multidirectional drug release, (2) bilayer systems with a drug-impermeable protective layer overlying a bioadhesive layer containing the drug, resulting in unidirectional release, and (3) “sandwich” structures of at least three layers that provide both unidirectional drug release and modified biphasic release by incorporating drugs into the different layers [9,10,180].

### 5.1. Oromucosal Patches

Mucoadhesive patches for application to the oral mucosa include polymer films, electrospun fiber-based materials, and 3D-printed structures; they can be either monolayer or multilayer systems, including sandwich structures [9]. Patches can be used for transmucosal drug delivery into the systemic circulation (buccal patches) as well as for local application (oromucosal patches). Patches can be cut to any size and applied directly to mucosal lesions. Buccal patches improve patient compliance compared to buccal tablets because they are physically flexible and, therefore, more comfortable for the patient [10,181].

Oromucosal patches are used in topical application for various oral diseases (ulcers, infections, oral lichen planus, etc.); they provide both effective treatment and protection of damaged tissues from various factors affecting pain receptors. Therefore, the use of oromucosal patches increases patient compliance, reduces pain, and improves quality of life [182]. The patch adheres securely to the oral mucosa through mucoadhesion. The multilayer structure of the patch allows for unidirectional drug release, which increases drug bioavailability and reduces toxicity and the incidence of side effects [108,183]. At present, mucoadhesive buccal multilayer patches are the most promising dosage form for use in the oral cavity [184]. For example, Tonglairoum et al. [185] developed multilayer oromucosal patches based on l-cysteine-thiolated chitosan, 2-hydroxypropyl-β-cyclodextrin (HPβCD), and polyvinylpyrrolidone (PVP). The resulting patches contained clotrimazole as an anti-inflammatory agent and were intended to improve the topical therapy of oral lichen planus and recurrent aphthous stomatitis. The clotrimazole-loaded PVP/HPβCD-based inner electrospun layer was coated with nanofibers composed of polyvinyl alcohol and chitosan or SH-chitosan. It was shown that SH-chitosan-coated patches exhibited better mechanical properties, greater flexibility and 1.5 times higher ex vivo mucoadhesion (porcine cheek pouch model) compared to chitosan-coated patches. The patches showed prolonged drug release for 8 h and improved antifungal activity against *Candida albicans* compared to commercial clotrimazole lozenges. Hydrophobic polymers such as ethylcellulose or polycaprolactone are also commonly used to provide a protective layer that allows unidirectional drug release [10,186,187].

A disadvantage of oromucosal patches in the form of biopolymer films and especially electrospun materials is the difficulty in providing and controlling prolonged drug release. An attractive approach for programming drug release could be the use of various nanofillers such as chitin and cellulose nanocrystals, bacterial cellulose nanofibers, and various technological techniques such as three-dimensional (3D) printing, cryogels, and aerogels formation [188,189,190,191]. For example, Olmos-Justea et al. [192] obtained an oromucosal patch with curcumin based on alginate and cellulose nanofibers by 3D printing, which were then sublimated to remove water and form porous aerogels. It was shown that 3–5% nanocellulose provided desirable viscoelastic properties for successful printing and high mechanical strength of the patches (Young’s modulus was 23–28 MPa, and compressive strength was more than 3 MPa). The obtained oromucosal patches had a high degree of swelling of about 1000–1200%. Moreover, by varying the amount of nanocellulose, the release rate could be controlled; for example, the release of curcumin from freeze-dried printed patches ranged from 100% in 6 h (3% cellulose nanofibers) to 50% in 24 h (5% cellulose nanofibers).

### 5.2. Buccal Tablets

Buccal tablets are a solid dosage form, usually obtained by direct compression (sometimes by wet granulation techniques), intended for application to the buccal mucosa. After application to the mucosa, buccal tablets should soften and maintain their position and geometric shape until complete drug release [193]. This dosage form is intended for systemic administration and should ensure absorption of the drug into the systemic bloodstream, bypassing the gastrointestinal tract, protecting the drug molecules from the aggressive environment of the stomach and enzymatic degradation, and increasing bioavailability due to the absence of hepatic first-pass metabolism [194]. To ensure unidirectional and sustained drug release and to improve mucoadhesion, buccal tablets can be coated with water-resistant mucoadhesive polymers such as ethylcellulose, etc. [195]. To regulate drug release, multilayer tablets can be prepared, and various drug-containing micro- and nanostructures (e.g., microspheres, etc.) can be incorporated into the tablet composition [196]. The main disadvantage of buccal bioadhesive tablets is insufficient flexibility and possible detachment from the mucosa with subsequent passage into the esophagus, which causes discomfort and reduces patient compliance [197].

### 5.3. Oromucosal Gels

Gels are semi-solid dosage forms that are solutions of substances converted into gel-like systems by various gel formers. Gels are easily dispersed on the oral mucosa and release the drug easily. Another advantage of gels is the simple technology of preparation. At the same time, gels have some significant disadvantages, including poorly controlled and multidirectional drug release and imprecise dosing compared to buccal tablets and patches.

Oromucosal gels are mainly used for topical application, the most common being dental gels with antimicrobial activity and analgesic and protective gels [198,199,200,201]. Various mucoadhesive gel compositions based on biopolymers such as chitosan, CMC, hyaluronic acid and xanthan gum are used to increase the residence time of the gel on the mucosa. Drug release from the gels can be tuned by varying the viscosity [202,203]. For example, Özdogan et al. [204] obtained chitosan-based gels with atorvastatin for the treatment of inflamed periodontal pockets. In vitro studies showed that the high mucoadhesive properties of chitosan gels ensured their prolonged retention at the site of application and prevented their rapid removal by saliva. In vitro tests (induced by tumor necrosis factor-alpha inflammation on the model of human gingival fibroblast cells) showed that the presence of chitosan in the gel composition enhanced the anti-inflammatory effect of atorvastatin. Hosny et al. [205] prepared a hyaluronic acid-based gel containing a nanoemulsion of miconazole. The in vitro study showed that the developed mucoadhesive composition prolonged the release of miconazole at the site of application and improved the penetration of miconazole into the tissues, thus reducing the dosage and frequency of drug administration and providing high efficacy in the therapy of oral candidiasis.

## 6. Nanotechnology-Based Systems to Improve Buccal Drug Delivery

The use of various types of nanoparticles is an advantageous strategy to improve buccal drug delivery by increasing the rate of drug diffusion across the mucosal and epithelial barrier, prolonging the residence time of the drug on the buccal mucosa, and protecting the drug from degradation in saliva [16,206]. Nanocarriers can provide modified and controlled drug release, reducing the frequency of administration and improving patient compliance [207,208]. The development of oromucosal formulations should take into account the aqueous nature of saliva, which forms a hydrophilic film on the mucosal surface, preventing the diffusion of lipophilic substances across the epithelium and allowing the absorption of hydrophilic compounds; therefore, nanoparticles based on or coated with hydrophilic biopolymers have increased permeability across the epithelium [197,209]. The mucoadhesive properties of biopolymers also influence permeability, e.g., positively charged or neutral particles have better mucoadhesion compared to negatively charged systems. As a result of prolonged and reliable contact with the mucosa, biopolymer particles provide a longer drug residence time and a higher drug dose at the site of administration. Penetration of drug-loaded biopolymer particles through the buccal epithelium, as well as free drug, can occur both transcellularly and paracellularly, although paracellular transport is most commonly used for larger particles. The level and rate of drug absorption from nanoformulations depend not only on the physicochemical properties of the biopolymer matrix but also on the type of nanocarrier, the presence of penetration enhancers and protease inhibitors in its composition.

The main types of nanoparticles used as buccal delivery systems are polymeric particles, solid lipid particles, liposomes, dendrimers, nanosponges, nanocrystals, hybrid particles, etc. [210]. anoparticles can usually be used as aqueous suspensions or can be incorporated into dosage forms such as gels, films, and even microneedles [211,212,213,214]. Overall, a commonly used technique is the incorporation of nanoparticles into various hydrogel systems [215,216,217,218]. Mucoadhesive properties of nanoparticles are achieved by using various biopolymers such as chitosan, sodium alginate, guar gum, hydroxyethyl cellulose, methylcellulose, etc. Cationic polymers, chitosan and chitosan derivatives are preferred over others to provide high mucoadhesion due to the formation of ionic bonds with negatively charged groups of mucin [219]. In general, the efficacy of nanoparticles as drug delivery systems depends on their physicochemical (nature of biopolymers, size, surface charge, polydispersity index), pharmaceutical (encapsulation efficiency and loading efficiency) and biopharmaceutical (modified release profile and prolonged residence time) characteristics [220].

The use of high surface area lipid-containing particles (liposomes and solid lipid particles) is a convenient strategy for the buccal bioavailability of hydrophobic drugs whose absorption is limited by their dissolution rate. The use of solid lipid nanoparticles is also an effective strategy for increasing the solubility and permeability of biopharmaceutical class II, III and IV drugs, resulting in increased drug bioavailability, controlled release and improved therapeutic efficacy [221,222]. Solid lipid particles are one of the most promising dosage forms for providing transport across biological membranes. Unlike liposomes, solid lipid particles are stable over long storage periods [223,224]. Solid lipid particles are often incorporated into buccal solid dosage forms. Le et al. [225] developed buccal tablets containing solid lipid particles with prednisolone. By varying the content of solid lipids and surfactants (including carbopol, stearic acid, and Tween 80), prolonged drug release ranging from 10 to 50% within 8 h was achieved. At the same time, the particle size varied from 90 nm to 4 μm. The permeability of the drug across the mucosa was influenced by both the content of solid lipids (less of them improved the wettability of the tablets and improved the permeability of the drug) and the size of the solid limiting particles (small nanoparticles of about 94 nm had the ability to penetrate the biological barrier, thereby enhancing the drug penetration). The ex vivo mucoadhesion time (porcine buccal pouch model) ranged from 3.5 to 6 h. Zewail et al. [221] used solid lipid particles to compress buccal tablets containing the non-steroidal anti-inflammatory drug lornoxicam. Lornoxicam has poor water solubility (class II according to the Biopharmaceutical Classification System) and a short half-life (3–4 h). The best formulation of lornoxicam-loaded solid lipid particles had a particle size of approximately 216 nm, an ζ-potential of −27 mV and an encapsulation efficiency of 93%. The resulting solid lipid particle-based tablets were characterized by a long mucoadhesion time and a controlled drug release profile. As a result, the resulting buccal tablets showed improved in vivo anti-inflammatory effects compared to free lornoxicam, including a significant early anti-inflammatory response at 1 and 2 h, as well as a prolonged effect at 4 h.

Hazzah et al. [226] developed lyophilized sponges based on solid lipid particles loaded with curcumin for buccal administration. For this purpose, curcumin was incorporated into solid lipid particles based on Gelucire and Poloxamer (hydrodynamic diameter was 300 nm and ζ-potential was about −15 mV), and then the resulting dispersion was incorporated into hydrogels based on different mucoadhesive polymers (such as Polycarbophil, HPMC, CMC, gellan gum, and sodium alginate) with the addition of plasticizer glycerol and cryoprotectant mannitol. Finally, the formed gels were lyophilized to obtain mucoadhesive buccal sponges. The results of the in vitro test showed that the lyophilized sponges exhibited extended sustained drug release of 10–20% over 6 h. The HPMC-based and polycarbophil-based sponges exhibited extended in vivo residence times of 4 and 15 h, respectively, providing therapeutic levels of curcumin in saliva.

It should also be noted that both hydrophobic and hydrophilic drugs can be incorporated into liposomes [227]. For example, Azim et al. [228] developed liposomes loaded with vitamin B6 to improve drug absorption and bioavailability and then incorporated them into a mucoadhesive buccal film based on HPMC and CMC. The resulting liposome-containing buccal film provided a prolonged release of vitamin B6 (73% in 6 h) compared to a liposome-free film (96% in 30 min). Ex vivo permeation analysis (chicken pouch model) showed that vitamin B6 within the liposome-containing film had a lower permeation rate across the membrane (37%) compared to both vitamin B6 within the liposome-free film and vitamin B6 solution.

Nano- and microparticles based on biopolymers such as cationic chitosan and anionic pectin, alginate and fucoidan in the form of polyelectrolyte complexes, including those cross-linked with sodium tripolyphosphate or zinc sulfate, have been widely used for the development of buccal drug delivery systems [127,229]. For example, Martín et al. [230] developed antifungal mucoadhesive systems based on biopolymer microparticles for the treatment of oral candidiasis. First, nystatin-loaded alginate microparticles were prepared by the emulsification/internal gelation method using an aqueous solution of sodium alginate, CaCO_3_, and nystatin as the internal phase and vegetable oil as the external phase. The microparticles were precipitated with calcium chloride and then additionally coated with low molecular weight chitosan. The resulting microparticles had hydrodynamic diameters ranging from 85 to 135 μM and ζ-potentials ranging from −40 mV (for alginate particles) to −8 mV (for chitosan-coated particles). In general, both types of microparticles had high mucoadhesive force; slightly higher mucoadhesion (about 1.2-fold) was inherent to chitosan-coated microparticles despite their low ζ-potential. The developed microparticles showed a prolonged release of nystatin of about 50–60% in 2.5 h. In vivo experiments showed that nystatin was retained in porcine mucosa at a concentration of 3–4 μg/cm^2^ after 1.5 h, whereas no nystatin was detected from a commercial suspension.

Hybrid nanoparticles composed of two or more materials (e.g., polymer+lipid, polymer+cellulose nanocrystals, polymer and inorganic fillers including metal/metal oxides, etc.) are attractive buccal drug delivery systems because they allow exploiting the advantages of different materials. Hybrid nanoparticles are able to provide high mucoadhesion, targeted delivery and modified release profiles through slow erosion of different layers [231,232,233]. For example, hybrid nanoparticles based on chitosan and clays (e.g., magnesium aluminosilicate, a mixture of montmorillonite and saponite clays) as cross-linking agents increase the stability of the system and prevent premature swelling and erosion of chitosan films, thereby prolonging drug release [234,235].

In other studies [236,237], the authors obtained buccal nanocomposite films based on oleic acid-modified starch containing carbon nanotubes hydrophilized by modification with D-glucose. Zolpidem was used as a model drug. It was shown that the surface modification of the nanotubes and their mixing with starch films led to an increase in their diameter from 27 nm to 46 nm while maintaining the homogeneity of the dispersions. The resulting nanocomposite exhibited prolonged release of zolpidem in 200 h compared to the zolpidem solution, which was completely released in 12 h.

Metal-containing hybrid nanoparticles are of interest as mucoadhesive drug platforms because metals or metal oxides have many useful properties. Metals have the ability to form various chemical bonds with polymers, and metals can also have intrinsic pharmacological activity (e.g., antitumor, antibacterial, wound healing, etc.). Incorporation of metals into the polymer matrix can improve its mechanical properties, retard erosion, and enhance biological effects [113,238,239]. For example, Mahmoud et al. [240] developed mucoadhesive hydrogel composites based on acrylic acid, polyethylene glycol and zinc oxide nanoparticles for local buccal delivery of propranolol. It was shown that the introduction of ZnO nanoparticles improved the thermal stability of the composites, but ZnO reduced the mucoadhesion of the obtained systems in artificial saliva solution about 2-fold; at the same time, it was possible to achieve high mucoadhesion by increasing the acrylic acid in the system up to 90 mass %. The developed composites provided a gradual drug release up to 9 h and also exhibited antimicrobial activity against *Staphylococcus aureus*, *Bacillus subtilis* and *Escherichia coli*.

In conclusion, different types of nanoparticles may be useful for use in oromucosal drug delivery because they allow the regulation of the targeting and profile of drug release, thereby minimizing systemic side effects and improving therapeutic efficacy.

## 7. Conclusions and Future Perspectives

Oromucosal (topical and buccal) drug delivery is a convenient and patient-friendly method of drug administration. Oromucosal drug delivery can protect the drug from enzymatic and acid degradation, pH and first-pass metabolism that occur in the gastrointestinal tract. Moreover, the use of oromucosal delivery systems allows to increase the bioavailability of drugs and reduce their systemic toxicity, especially the local oromucosal application of various substances used in the treatment of oral cavity pathologies of inflammatory and immunogenic genesis (e.g., corticosteroids). To realize their advantages, oromucosal drug delivery systems must meet certain requirements, in particular (i) high mucoadhesion (to reliably prolong the residence time of the drug on the mucosa), (ii) programmable and controlled release profile, and (iii) enhanced permeability across the mucosal membrane.

Biopolymers and their derivatives are a good choice for the R&D of oromucosal drug delivery systems. In particular, we highlight the excellent prospects of a new class of mucoadhesive polymers modified with catechol-containing moieties. Mussel-inspired chemistry is a promising way to modify biopolymers for biomedical purposes, as it allows both a significant enhancement of their mucoadhesion and opens a wide possibility for coupling/complexation of drug molecules and bioactive metals.

Biopolymer-based systems (including solids, tablets and patches, semi-solids, hydrogels and liquid polymer solutions) are ideal candidates for oromucosal drug delivery. We believe that oromucosal patches are the most promising because they can provide unidirectional drug release, thereby increasing both local and systemic bioavailability and reducing toxicity and the incidence of side effects. Biopolymers are non-toxic, biocompatible and biodegradable materials; biopolymers are characterized by high mucoadhesion and can enhance the drug permeability across biological membranes.

The main problem with the use of oromucosal buccal drug delivery systems is the low transbuccal permeability of drugs. Some excipients such as protease inhibitors and permeability enhancers can be used to improve the bioavailability of buccally administered drugs, and the current trend to improve the properties of existing oromucosal drug delivery systems is the use of various nanoparticles (solid lipid particles, biopolymer particles, hybrid particles, etc.) which can be incorporated into oromucosal tablets, patches or gels to provide their superior permeability across the buccal epithelium.

We believe that nanoparticles are the most promising structures for creating formulations that can effectively deliver drugs through the buccal route. We believe that in the coming years, more research will be devoted to the development of oromucosal drug delivery systems, including nanotechnology-based systems, for various therapeutic applications. The primary focus of these studies should be to improve buccal drug permeation without developing systemic toxicity for buccal dosage forms and to ensure strictly unidirectional drug release for topical oromucosal dosage forms. Such developments are an important step in the pharmaceutical R&D of new, safe and effective drugs.

## Figures and Tables

**Table 1:** Mucoadhesive biopolymers.

Biopolymers	Mucoadhesive Properties
Cationic (such as chitosan and its cationic derivatives)	Chitosan is characterized by high mucoadhesion due to the formation of ionic bonds with mucins. Cationic quaternized derivatives of chitosan are characterized by improved mucoadhesion compared to chitosan and also form more stable polyelectrolyte complexes with various polyanions. Chitosan and its derivatives improve the intercellular permeability of drugs by opening up tight junctions.
Anionic (such as alginic acid, hyaluronic acid, pectin, CMC, carrageenans, fucoidans)	Anionic polymers have high mucoadhesive properties due to the formation of strong hydrogen bonds with mucins. Moreover, the presence of carboxyl or sulfate functional groups with different pKa allows us to vary the properties of drug delivery systems based on them, especially the stability of the complexes and the rate of drug release.
Non-ionic (such as starch, cellulose, HPMC, methylcellulose)	In general, the mucoadhesive properties of nonionic biopolymers should be improved by grafting fragments that enhance mucoadhesion.
Polyampholites (such as collagen, gelatin)	The mucoadhesive properties of polyampholytes can be controlled by changing the pH.
Thiolated biopolymers	Thiolated polymers have higher mucoadhesive properties due to the formation of a disulfide bridge with mucin molecules.
Catechol-based biopolymers	Catechol moieties provide extremely strong adhesion, even in wet environments, as well as broad possibilities for conjugation with bioactive molecules. Catechol moieties can be formed by additional coordination bonds, π-π stacking, and π-cation interactions.
